# Muscle Uncoupling Protein 3 Expression Is Unchanged by Chronic Ephedrine/Caffeine Treatment: Results of a Double Blind, Randomised Clinical Trial in Morbidly Obese Females

**DOI:** 10.1371/journal.pone.0098244

**Published:** 2014-06-06

**Authors:** Renata Bracale, Maria Letizia Petroni, Sergio Davinelli, Umberto Bracale, Giovanni Scapagnini, Michele O. Carruba, Enzo Nisoli

**Affiliations:** 1 Department of Medicine and Health Sciences, University of Molise, Campobasso, Italy; 2 Clinical Nutrition Laboratory, IRCCS Institute Auxologico Italiano, Piancavallo (Verbania), Italy; 3 Inter-University Consortium “SannioTech”, Benevento, Italy; 4 Department of Public Health, University of Naples “Federico II”, Naples, Italy; 5 Center for Study and Research on Obesity, Department of Medical Biotechnology and Translational Medicine, University of Milan, Milan, Italy; Weill Cornell Medical College Qatar, Qatar

## Abstract

Ephedrine/caffeine combination (EC) has been shown to induce a small-to-moderate weight loss in obese patients. Several mechanisms have been proposed, among which an increased thermogenic capacity of skeletal muscle consequent to the EC-induced up-regulation of uncoupling protein 3 (UCP3) gene expression. We did a parallel group double-blind, placebo-controlled, 4-week trial to investigate this hypothesis. Thirteen morbidly obese women (25–52 years of age, body-mass index 48.0±4.0 kg/m^2^, range 41.1–57.6) were randomly assigned to EC (200/20 mg, n = 6) or to placebo (n = 7) administered three times a day orally, before undergoing bariatric surgery. All individuals had an energy-deficit diet equal to about 70% of resting metabolic rate (RMR) diet (mean 5769±1105 kJ/day). The RMR analysed by intention to treat and the UCP3 (long and short isoform) mRNA levels in rectus abdominis were the primary outcomes. Body weight, plasma levels of adrenaline, noradrenaline, triglycerides, free fatty acids, glycerol, TSH, fT4, and fT3 were assessed, as well as fasting glucose, insulin and HOMA index, at baseline and at the end of treatments. Body weight loss was evident in both groups when compared to baseline values (overall −5.2±3.2%, p<0.0001) without significant differences between the treated groups. EC treatment increased the RMR (+9.2±6.8%, p = 0.020), differently from placebo which was linked to a reduction of RMR (−7.6±6.5%, p = 0.029). No significant differences were seen in other metabolic parameters. Notably, no changes of either UCP3 short or UCP3 long isoform mRNA levels were evident between EC and placebo group. Our study provides evidence that 4-week EC administration resulted in a pronounced thermogenic effect not related to muscle UCP3 gene expression and weight loss in morbidly obese females under controlled conditions.

**Trial Registration:**

ClinicalTrials.gov NCT02048215

## Introduction

Ephedrine and caffeine (EC) combination has been widely used in human obesity treatment [1], [2], and is still present in many herbal preparations sold widespread in many countries for weight loss. It is well known that this drug increases the metabolic rate in both animals and humans [3], [4]. Ephedrine is an agonist of both α- and β-adrenoceptors; moreover, it induces noradrenaline release from sympathetic neurons, and thus it is a sympatho-mimetic drug with a mixed profile [5]. Caffeine increases both noradrenaline and dopamine release and stimulates the neuronal activity in several brain regions. In addition, caffeine antagonizes the inhibitory effects of adenosine on sympathetic nervous system (SNS). This modulation of SNS activity may be a possible explanation for the thermic effect of EC [6], [7]. In fact, noradrenaline activates the uncoupling protein 1 (UCP1), a member of mitochondrial carriers localized on the inner mitochondrial membrane in brown adipocytes [8]. The physiological role of UCP1 is to uncouple oxidative phosphorylation, therefore most of the energy is dissipated as heat rather than being converted to ATP [9].

In addition to UCP1, expressed exclusively in brown adipose tissue (BAT), where it plays an important role in adaptive thermogenesis and energy expenditure in rodents and possibly in humans [10], [11], two other members of the mitochondrial anion carrier protein family play important physiological role. UCP2 is widely expressed in human tissues, including skeletal muscle, fat, heart, placenta, lung, liver, kidney, and pancreas, where it is involved in the control of radical oxygen species (ROS) production [12], [13]. UCP3 is expressed almost exclusively in skeletal muscle [14] and although its function is still not clearly established, therein it would be involved in decreasing ROS production and promoting muscle fatty acid oxidation [15]. Unlike UCP1 and UCP2, the UCP3 exhibits two transcriptional isoforms: a long form (UCP3L) and a short form (UCP3S). Clapham et al. [16] showed that transgenic mice overexpressing UCP3 were lean, despite the fact that they were hyperphagic, in comparison to their wild-type littermates. The 66-fold up-regulation of UCP3 mRNA in skeletal muscle was linked to improved glucose tolerance, decreased fasting blood glucose and insulin levels, 25% increase in resting oxygen consumption, decreased total cholesterol, decreased fasting blood glucose and insulin levels, and a 44% to 57% reduction in adipose tissue over total animal volume [16]. Moreover, 5 days on caloric restriction resulted in ∼2- to 3-fold increase of the UCP3 mRNA levels in lean and obese humans [17], and caffeine upregulates UCP3 expression in skeletal muscle, which was suggested to contribute to thermogenesis in obese yellow KK mice [18]. Aims of the present work were to investigate changes in energy expenditure and UCP3 expression in skeletal muscle of morbidly obese females treated with either placebo or EC.

## Materials and Methods

### Ethics Statement

The trial was done in accordance with the Declaration of Helsinki and ICH Good Clinical Practice. The protocol was approved by Istituto Auxologico Italiano and Regione Piemonte (Italy) Institutional Review Boards, and participants provided written informed consent. The protocol for this trial and supporting CONSORT checklist are available as supporting information; see Checklist S1 and Protocol S1. The trial started (first patient enrolled) in February 2000 and ended (last patient completed the clinical study) in January 2001. The study was not registered in a Clinical Trial Registry since enrolment of participants started before 2005: at that time this was neither requested by National Authority nor was an established procedure [19]. This has been done in retrospect at ClinicalTrials.gov (Identifier: NCT02048215). The authors confirm that all ongoing and related trials for this drug intervention are registered.

### Patients and Study Design

This was a randomized double-blind, parallel group study. Morbidly pre-menopausal obese female patients, non-smokers or smoking less than 5 cigarettes per day, were selected from the waiting list for bariatric surgery at Department of Surgery (Molinette Hospital, Turin, Italy). In all patients the body weight was stable during the three months before the study. To be eligible for surgery the inclusion criteria were: BMI ≥40 kg/m^2^, unresponsiveness to previous medically supervised weight loss programs, and no major psychiatric disorders.

The exclusion criteria were: pregnancy, ischaemic heart disease, cardiac failure, high blood pressure requiring drug treatment, tachyarrhythmia, sick sinus syndrome, atrioventricular block, two-bundle ventricular block, cerebrovascular diseases, occlusive peripheral artery disease, renal failure, and current treatment with drugs that might affect metabolic rate (e.g. β-adrenergic blockers, thyroid hormones). According to previous studies about the effect of the EC combination on thermogenesis in humans [20] and rhesus monkeys [21], we expected a minimal difference in resting metabolic rate (RMR) between treated and untreated subjects not inferior to 17%, and mean to standard deviation ratio not inferior to 10. Therefore, a minimum sample size of 12 subjects (6 in each treatment group) was considered adequate for a power of 0.8 and alpha level of 0.05. Patients were randomised to 28-day treatment with either EC (200/20 mg ter in die [t.i.d.]) or placebo. Allocation ratio was 1∶1, with a block size of 4 (i.e. every four patients two were allocated to EC and two to placebo). Masking was double blind (subject, caregiver, investigator, outcomes assessor). Randomisation lists was prepared – using standard randomization number generation techniques – by the statistics consultant of the Istituto Auxologico Italiano who had no contact with patients. The randomization list was passed to the Hospital Pharmacy which supplied the active compounds and placebo, and which prepared sequentially numbered containers of the study pills. The principal clinical investigator (MLP) enrolled participants, and assigned participants to interventions. The EC administration started with an initial dose of 100/10 mg ter in die, for the first week and then proceeded with the full dose of 200/20 mg t.i.d. In order to evaluate drug safety, the blood pressure was measured three times a day; both electrocardiography and echocardiography were recorded at baseline and every week. Body weight was measured in a standardised manner at start and at the end of treatment. During the treatment period all patients were fed a hypocaloric diet (total energy content of ∼70% of energy expenditure, as measured by indirect calorimetry), and containing 20% proteins, 55% carbohydrates, 25% fat half of which was monounsaturated, and 35 g/day fibres. They were hospitalised, during the whole treatment period, at the metabolic unit of San Giuseppe Hospital-Istituto Auxologico Italiano at Piancavallo (Verbania, Italy). After diet and drug period, the patients were transferred to the Department of Surgery (Molinette Hospital, Turin) for the bariatric surgery. The drug treatment (EC and placebo) was stopped the day before surgical intervention. Small biopsies of rectus abdominis were taken during surgery, immediately frozen in liquid nitrogen, and stored at −80°C for subsequent analysis.

Primary clinical outcome was the change in resting metabolic rate (RMR), while the primary non-clinical outcome was the UCP3 gene expression in skeletal muscle. Secondary outcomes were changes in body weight and metabolic parameters (see below), and drug safety.

### Clinical Chemistry

After an overnight fast, blood was sampled to measure glucose, insulin, free-fatty acids, glycerol, thyroid hormones, noradrenaline. RMR was measured by indirect calorimetry. Plasma insulin levels were measured by using a commercial enzymatic method (Tosoh, Kyobashi Chuo-Ku, Tokio; Japan). Plasma samples for adrenaline and noradrenaline levels were collected in refrigerated vials containing EGTA and glutathione, stored at −80°C and subsequently analysed by high-performance liquid chromatography [22]. FFA were measured by an enzymatic assay (NEFA C, Wako Chemicals GmbH, Japan). Glycerol was measured by a spectrophotometric method [23]. All other determinations were carried out by standard automated procedures. Homeostasis model assessment (HOMA) was calculated as fasting plasma glucose (mmol/l)/fasting plasma insulin (mU/l) [24].

### Resting Metabolic Rate Measurement

Each subject, after an overnight fast (12–14 hours) and 30 minutes of resting, underwent RMR measurement: expired gases were collected using a ventilated hood calorimeter (Sensor Medics 2900, Anaheim, CA). The O_2_ and CO_2_ analysers were calibrated before each test using gases of known O_2_ and CO_2_ concentration. Room temperature was maintained between 23°C and 26°C throughout the study and all precautions were taken to avoid any disturbing factors that could affect metabolic rate. The RMR represents an average of the steady state (SS) periods. SS was defined as a variation of not more ±5% in VO_2_ and VCO_2_ for at least 5 minutes. RMR was calculated with the Weir formula [25].

### Quantitative Polymerase Chain Reaction

Total RNA was isolated from skeletal muscle using the RNeasy Lipid Tissue Kit (Qiagen, Milan, Italy). RNA was incubated with DNase I and eluted in RNase-free water. The concentration of RNA was determined by absorbance at 260 nm. Total RNA was reverse transcribed (1 µg) using iScripTM cDNA Synthesis Kit (Bio-Rad Laboratories, Italy). Specific sense and antisense primers were designed using Beacon Designer 2.6 software from Premier Biosoft International (Palo Alto, CA, USA) and synthesized by PRIMM (Milan, Italy).

PCR was performed using the following primers: UCP3 short form: sense primer GGACTATGGACGCCTACAGAAC, antisense primer GGAAGTTGTCAGTGAGCAGGTG; UCP3 long form: sense primer CCATCGCCAGGGAGGAAGG, antisense primer GGAAGTTGTCAGTGAGCAGGTG; housekeeping gene 18S: sense primer CTGCCCTATCAACTTTCGATGGTAG, antisense primer CCGTTTCTCAGGCTCCCTCTC. Triplicate PCR reactions were carried out with the intercalating dye SybrGreen. A melting curve was recorded at the end of PCR to ensure the specificity of amplicons. Each sample (25 µl) contained 12.5 µl iQ™ SybrGreenI SuperMix (Bio-Rad), 0.4 µM each primer and 1/50 of reverse transcriptase product. PCR cycles were programmed on an iCycler iQ™ Real Time PCR detection system (Bio-Rad). The cycle number at which the various transcripts were detectable (CT) was compared to that of 18S, referred to as ΔCT. The gene relative level was expressed as 2^−(ΔΔCT)^, in which ΔΔCT equals ΔCT of the treated minus ΔCT of the controls.

### Statistical Analysis

In order to compare differences between the two treatment groups at baseline Student’s t test for independent samples, and a preliminary Kolmogorov-Smirnov test were performed on all the biochemical and clinical parameters considered in the study. Since we confirmed all data were distributed in a parametric fashion (p>0.05 at Kolmogorov-Smirnov test), only paired or unpaired (as adequate) Student’s t test was employed for analysing the data. Comparison of outcomes between baseline and following the 28-day treatment was carried out by Student’s t test for paired samples. Statistical analysis was performed by Microsoft Excel and SPSS software (SPSS Inc, Chicago, Illinois, USA).

## Results

**Figure 1:**
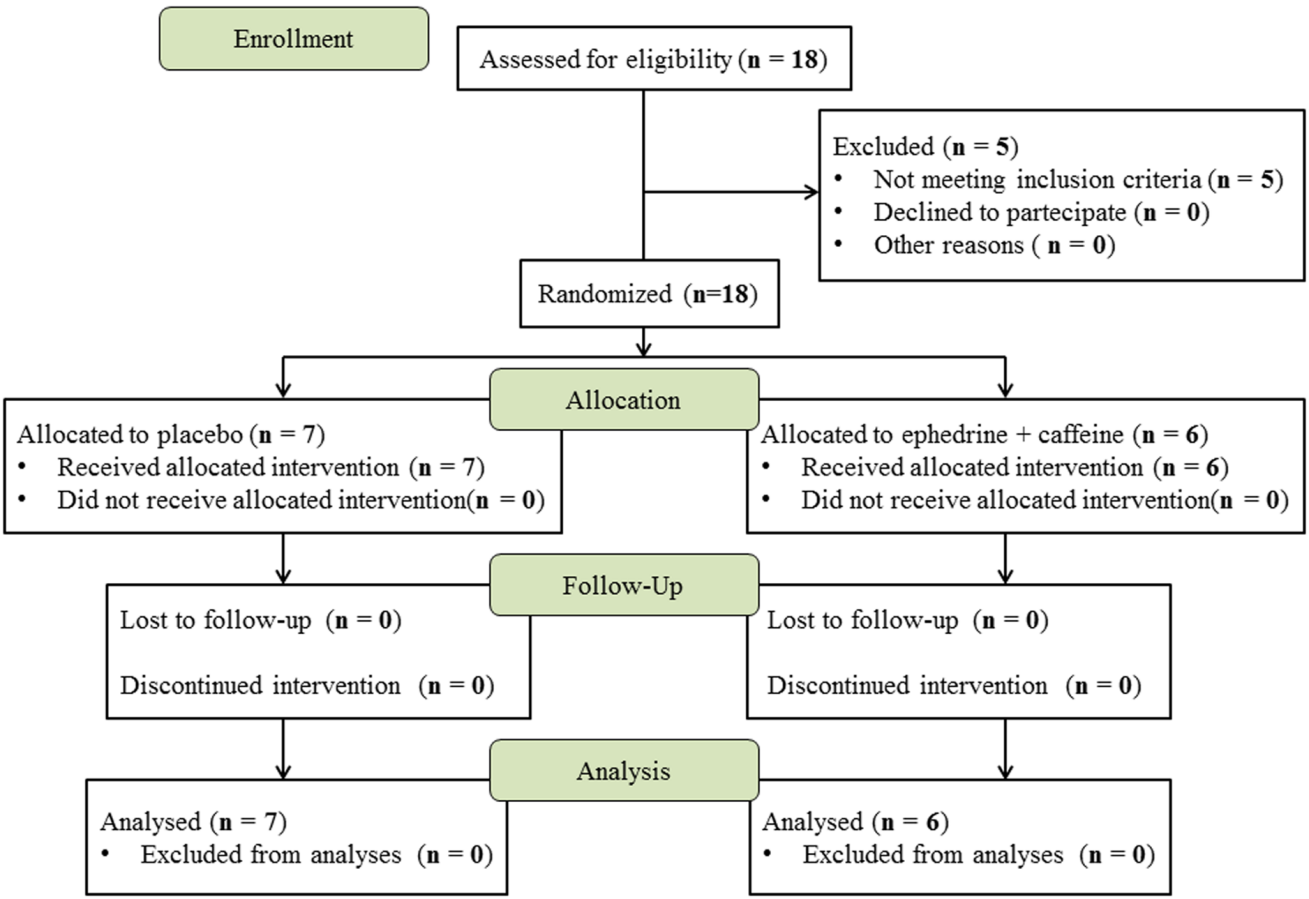
Flow diagram of patients’ enrollment and follow up.

The flow diagram of patients’ enrollment and follow up is reported in Figure 1. Out of eighteen patients screened, five were excluded (all because of diabetes being diagnosed at baseline). We studied 13 morbidly obese females, 36.3±10.3 year-old (mean ± SD; range 25–52 years) with body mass index (BMI) by 48.0±4.0 kg/m^2^ (mean ± SD; ranging from 41.1 to 57.6 kg/m^2^). All enrolled patients completed the study. No cardiovascular side effects occurred over the study period. Three patients (one in placebo and two in EC group) complained of insomnia after the first week and were returned to half dosage for the entire treatment period. Following the 28 day-treatment period, there was a significant weight loss in both treatment groups vs. baseline (overall −5.2±3.2%, p<0.0001) as well as a reduction in BMI value (Table 1), with no differences between the two groups. EC increased the RMR (+9.2±6.8%, p = 0.020), whereas a significant decrease of RMR was evident in the placebo group (−7.6±6.5%, p = 0.029) (Table 1). Patients receiving EC treatment showed a mild increase in serum creatinine relative to placebo group, but in none of patients this exceeded normal ranges. Circulating alanine transaminase levels were slightly increased in the EC group following treatment. There was no significant difference between the two treatments in the other measured biochemical parameters (Table 1).

**Table 1:** Anthropometric, biochemical, energy expenditure, and metabolic parameters.

	EC				Placebo		
	*p* ^a^	Mean	SD	*p^b^*	Mean	SD	*p* ^a^
Weight (kg)	0.001	123.67	15.63	0.71	120.50	14.08	0.002
		117.28	14.84	0.35	110.46	7.37	
BMI (kg/m^2^)	0.009	49.10	4.80	0.35	46.76	3.63	0.022
		46.03	3.61	0.41	44.28	2.79	
Waist (cm)	0.142	132.33	12.96	0.77	134.33	10.05	0.082
		128.00	13.58	0.82	126.40	9.04	
Calorie Intake during treatment (kcal)	0.363	1417	387	0.73	1357	152	0.661
		1425	376	0.4	1277	100	
RMR (kcal/24 h)	0.02	2060	182	0.34	1938	243	0.029
		2243	132	0.011	1791	260	
Adrenaline (pg/mL)	0.345	28.83	13.91	0.51	24.83	1.47	0.892
		26.60	12.66	0.86	25.40	6.23	
Noradrenaline (pg/mL)	0.752	263.83	127.21	0.65	229.00	128.68	0.979
		297.67	187.24	0.52	239.40	85.52	
ACTH (pg/mL)	0.442	46.74	33.63	0.78	41.57	27.09	0.319
		35.95	19.41	0.35	24.15	11.63	
Cortisol (µg/mL)	0.292	18.25	13.27	0.96	18.60	12.40	0.115
		14.50	6.76	0.29	10.16	5.11	
HOMA	0.549	3.99	1.16	0.65	3.65	1.43	0.695
		3.68	1.17	0.5	3.23	0.95	
Insulin (µU/mL)	0.61	18.43	5.93	0.49	16.04	6.18	0.279
		17.33	2.78	0.82	16.62	6.17	
C-peptide (ng/mL)	0.361	4.13	0.78	0.23	3.51	0.95	1.00
		3.80	0.60	0.53	3.40	1.06	
Creatinine (mg/dL)	0.013	0.85	0.29	0.23	0.69	0.11	0.53
		0.97	0.29	0.11	0.73	0.14	
AST (U/L)	0.064	21.67	5.89	0.14	17.29	3.15	0.14
		25.00	8.74	0.98	24.83	10.05	
ALT (U/L)	0.028	28.17	12.67	0.98	28.00	11.83	0.215
		37.50	19.99	0.65	44.50	30.38	
Total cholesterol (mg/dL)	0.061	236.17	37.05	0.16	208.57	27.01	0.171
		202.00	20.21	0.46	192.17	24.33	
HDL cholesterol (mg/dL)	0.603	48.83	15.17	0.38	42.43	7.11	0.43
		44.83	11.11	0.47	40.67	7.61	
Triglyceride (mg/dL)	0.83	181.50	58.31	0.35	151.43	50.73	0.68
		177.67	31.92	0.27	149.67	48.62	
TSH (µU/mL)	0.181	3.34	2.85	0.21	1.63	0.74	0.904
		1.99	0.90	0.43	1.54	0.98	
fT3 (ng/dL)	0.246	2.73	0.42	0.73	2.63	0.57	0.355
		3.10	0.72	0.42	2.76	0.56	
fT4 (ng/dL)	0.173	10.57	2.31	0.07	12.99	1.85	0.283
		11.47	1.59	0.07	13.68	2.16	
Glycerol	0.642	0.32	0.16	0.39	0.41	0.13	0.553
		0.34	0.15	0.74	0.32	0.00	
FFA	0.364	906.83	366.76	0.52	771.75	271.48	0.821
		721.17	246.91	0.94	731.33	157.60	

For each parameter the above value refers to basal untreated value (T_0_), while the bottom value refers to the value after treatment (T_1_). ^a^T_1_ *vs*. T_0_ comparison by Student’s t test (paired data); ^b^EC group vs placebo group comparison by Student’s t test (unpaired data). BMI, body mass index; RMR, resting metabolic rate; ACTH, adrenocorticotropic hormone; HOMA, homeostatic model assessment; AST, aspartate aminotransferase; ALT, alanine transaminase; HDL, high-density lipoprotein; TSH, thyroid-stimulating hormone; fT3, free T3 (the active part of triiodothyronine); fT4, free T4 (the active part of thyroxine); FFA, free fatty acids. Microgram (µg); microunits (µU).

**Figure 2:**
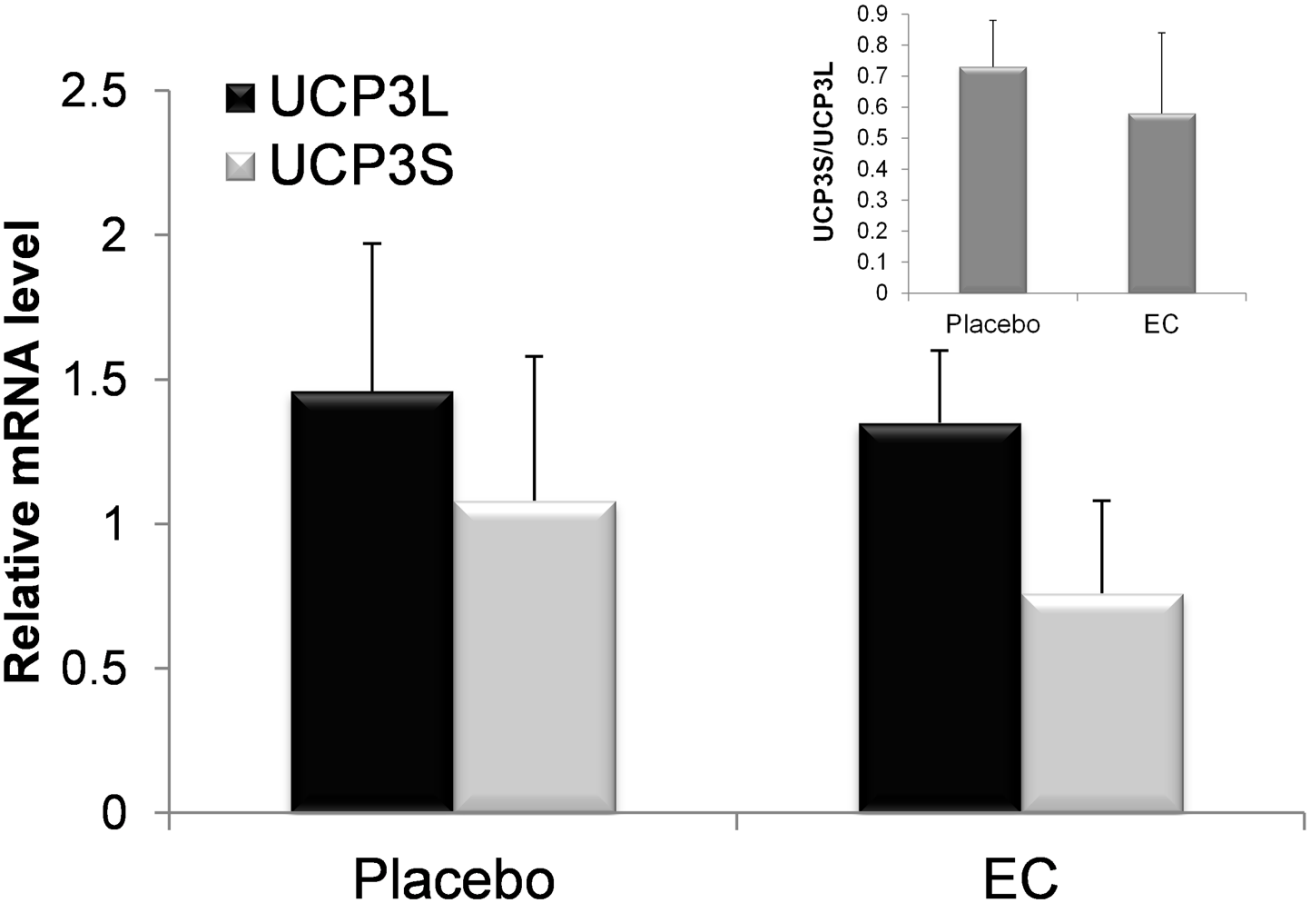
UCP3 isoform mRNA levels in skeletal muscle of placebo- and EC-treated morbidly obese women. UCP3 long (UCP3L) and short (UCP3S) mRNAs were measured by quantitative RT-PCR. mRNA levels were normalized to 18S RNA. n = 6 or 7 patients assigned to EC or to placebo, respectively; error bars, s.d. The inset shows the UCP3S/UCP3L mRNA ratio. The PCR experiments were performed twice in triplicate. Data were analysed by unpaired Student’s t test.

Relative levels of UCP3S and UCP3L mRNAs were measured by quantitative RT-PCR in the skeletal muscle samples. Figure 1 shows that no statistically significant changes of both short and long UCP3 was evident between the two groups of patients. Likewise, UCP3S/UCP3L mRNA ratio was not affected by the EC treatment (Figure 2, inset). Thus, under our experimental conditions the EC treatment was unable to change the muscle UCP3 mRNA levels compared to placebo in morbidly obese females.

## Discussion

Because only a few small studies have been done in humans to investigate the thermogenic effects of EC, in the present study we examined the UCP3 expression in skeletal muscle of pre-menopausal morbidly obese females treated with either placebo or EC for 28 days. Our findings demonstrate that chronic treatment with EC increased the RMR, in contrast with placebo which decreased the RMR in obese patients. This result is consistent with a previous study showing that the decrease in 24-hour energy expenditure seen in the placebo group was 10% at day 1 and 13% at day 56, but was only 7% and 8% in the EC-treated group, and the weight-loss was not different in the two groups after 8-week treatment [26]. On the other hand the results from placebo controlled studies, conducted in different clinical settings and in several countries, were highly consistent that EC is an effective and well tolerated anti-obesity therapy [27]–[33]. Several reasons may justify this discrepancy. First, our is the only study conducted in patients with very high grade obesity addressed to bariatric surgery. Accordingly, one month may be a too short treatment period to obtain relevant anti-obesity results, particularly in patients with a massive obesity characterized by high sympathetic activity [34], [35]. In addition, our study was performed in a small sample of patients, and this might have limited the chance to observe the drug efficiency.

The reported increase of RMR after EC treatment may be due to different physiological mechanisms, including an increased energy expenditure through thermogenesis of brown adipose tissue (BAT) and skeletal muscle [35]. At present, the recruitment in BAT (i.e., the process including cell proliferation, mitochondrial biogenesis, and increases in functional mitochondrial UCP1 content) is considered of great interest. This is because BAT is now considered an active tissue even in adult humans [36], with the capacity to oppose obesity or its development by burning some of the energy we consume by feeding. In particular, human BAT depots would be constituted mainly of beige/brite adipocytes [37], expressing UCP1 when physiologically stimulated by cold or drugs. Thus increasing proliferation and activation of these fat cells might play a relevant role in obesity treatment. However, a more appropriate reevaluation of these findings suggest that the relative contribution of the beige/brite adipose tissue to the total thermogenesis capacity, at least in animals, would be marginal [38].

We focused our attention to the putative thermogesis activity of skeletal muscle by investigating UCP3 expression in morbidly obese females. We found no changes in UCP3S and UCP3L isoform mRNAs in rectus abdominis of obese treated with EC in comparison to obese patients treated with placebo. These results would suggest that muscle UCP3 is not directly linked to the increased RMR induced by EC in obese subjects. A relevant limitation of our study is that we could measure muscle UCP3 expression only as mRNA, but not as protein. Rephrasing the title of a thoughtful review article by Nedergaard and Cannon [38] “UCP3 mRNA does not produce heat”. On the other hand our conclusion is supported by data obtained by Nabben et al. [15] in skeletal muscle of UCP3^−/−^ mice, and showing no evidence for the function of UCP3 being basal or induced uncoupling.

Thus, how does EC treatment increase RMR value in obese women? It has been shown that ephedrine stimulates brown adipocyte respiration via β-adrenoceptors [39], and that the thermogenic action of ephedrine can be enhanced by methylxanthines, such as caffeine, through their ability to inhibit the phosphodiesterase-induced degradation of intracellular cyclic AMP [40], and to antagonize the inhibitory action of adenosine [41]. Anyway, several human studies have shown positive effects on body-weight management of EC [42]. Besides activating β_2_-adrenoceptors, EC treatment was found to enhance the β_3_-adrenoceptor expression in white adipocytes of obese subjects, following hypocaloric diet, when compared to placebo [43]. Together, those and our results allow to speculate that long-term treatment with EC further increases the already high level of SNS activity in morbidly obese patients. No significant changes in the studied biochemical parameters were observed, and only a mild increase in creatinine level, a reliable indicator to estimate skeletal muscle mass status, was detected. We did not find any other considerable variations, and the differences between the two groups were not statistically significant. These data would strengthen the safety profile of EC in morbid obesity, although during the last years there have been raising safety concerns about the use of ephedrine for pharmaceutical preparations, and the combination (expecially in botanical formulations) was banned by the U.S. Food and Drug Administration [44].

## Supporting Information

Checklist S1 **CONSORT Checklist.**
(DOC)

Protocol S1 **Trial Protocol.** Evaluation of diet and treatment with a combination of ephedrine and caffeine on thermogenesis, cardiac function and on uncoupling proteins expression in adipose and muscle tissue of morbid obese patients undergoing bariatric surgery.
(DOC)
