# Supplementary material for: Muscle Uncoupling Protein 3 Expression Is Unchanged by Chronic Ephedrine/Caffeine Treatment: Results of a Double Blind, Randomised Clinical Trial in Morbidly Obese Females
Source: PLoS One. 2014 Jun 6;9(6):e98244. doi: 10.1371/journal.pone.0098244 (PMC4048162; doi:10.1371/journal.pone.0098244)
Supplement: Protocol S1 — Trial Protocol. Evaluation of diet and treatment with a combination of ephedrine and caffeine on thermogenesis, cardiac function and on uncoupling proteins expression in adipose and muscle tissue of morbid obese patients undergoing bariatric surgery. (DOC) [file pone.0098244.s002.doc]

***Finalized research 1999 Istituto Auxologico Italiano for the Ministry of Health “Interaction between nutritional, social-behavioral and metabolic factors for prevention of cardiovascular disease: development of nutritional strategies on general population”.***

**Subproject 4.** Clinical Nutrition Laboratory (Professor Franco Balzola).

EVALUATION OF DIET AND TREATMENT WITH A COMBINATION OF EPHEDRINE AND CAFFEINE ON THERMOGENESIS, CARDIAC FUNCTION AND ON UNCOUPLING PROTEINS EXPRESSION IN ADIPOSE AND MUSCLE TISSUE OF MORBID OBESE PATIENTS UNDERGOING BARIATRIC SURGERY.

Main Investigator: Maria Letizia Petroni, MD

Collaborations:

- Professor Saverio Cinti, Chair of Human Anatomy, University of Ancona
- Professor Michele Carruba, Chair of Pharmacology, University of Milan
- Dr. Mauro Toppino, Surgical Department, Molinette Hospital, Turin

**Background**

Obesity is characterized by an imbalance between energy intake and expenditure, resulting in a net increase in the storage of body energy primarily as fat. This imbalance is the result of either a high energy intake, low energy expenditure, or a mixture of the two conditions. The administration of ephedrine and caffeine (E+C) has been proposed to promote weight loss by increasing energy expenditure and decreasing food intake. Ephedrine has been shown to increase energy expenditure in humans (1). The mechanism for this effect appears to be related to stimulation of release of norepinephrine (2) and direct agonistic stimulation of adrenoreceptors (3). Both of these actions may stimulate energy expenditure through increases in intracellular concentrations of cyclic AMP. The ephedrine-induced stimulation of energy expenditure is further enhanced when ephedrine is administered in combination with methylxanthines such as caffeine (4). The mechanism for methylxanthine enhancement of ephedrine is likely the result of inhibition of phosphodiesterase enzyme activity and antagonism of the inhibitory effect of adenosine on norepinephrine release (5). A study in postobese and lean volunteers first showed in humans that E+C was more effective than ephedrine alone at raising energy expenditure and E+C could effectively raise energy expenditure in individuals predisposed to obesity (6). Several studies have shown that E+C can effectively cause weight loss in humans (7-9). The contribution of increased energy expenditure or decreased food intake to weight loss with E+C treatment is still not firmly established, with studies report-Indeed, ephedrine and caffeine (EC) combination has been registered and approved as current treatment for weight loss in Denmark (Letigen®) since 1990. The mechanisms through which this combination works at cell level are yet not fully explained. It is known that epinephrine activates the uncoupling protein 1 (UCP1), a member of mitochondrial carriers localized on the inner mitochondrial membrane in brown adipocytes (10). The physiological role of UCP1 is to uncouple oxidative phosphorylation, therefore most of the energy is dissipated as heat rather than being converted to ATP. It has been shown that the cold-induced occurrence of brown-like adipocytes and UCP1 requires the presence of the β3-adrenoceptor in previously white adipose tissue and the presence of the β3-adrenoceptor is required for full stimulation of energy expenditure and oxygen consumption in white adipose tissue (11).  In addition to UCP1, expressed exclusively in brown adipose tissue (BAT), another member of the mitochondrial anion carrier protein family i.e. uncoupling protein 3 (UCP3) could play a physiological role in energy homeostasis. It is expressed almost exclusively in skeletal muscle (12) and exhibits two transcriptional isoforms: a long form (UCP3L) and a short form (UCP3S). A brief caloric restriction resulted in ~2- to 3-fold increase in UCP3 mRNA levels in lean and obese humans (13).

No data is available on the effect of the ephedrine+ caffeine combination on expression of β3-adrenoceptor in human adipose tissue and on UCP-3 expression in human muscle.

**Aim of the study**

To study changes in energy expenditure and to correlate them with UCP3 expression in skeletal muscle and the beta-3 adrenoceptor expression in adipose tissue of pre-menopausal morbidly obese females treated with either placebo or the combination of ephedrine and caffeine - at dosages already appoved by regulatory authorities in Denmark for weight loss in obesity - for 30 days prior to bariatric surgery.

**Patients and methods**

Adult females with morbid obesity, and in whom a weight loss prior to surgery is clinically indicated to reduce surgical and anaesteriological risk, will be enrolled from the waiting list for bariatric surgery at Department of Surgery of Molinette Hospital at Turin. Those who will agree to take part in the protocol following written informed consent will be hospitalised at the Metabolic unit of San Giuseppe Hospital in Piancavallo for four weeks prior to surgery.

Inclusion Criteria:

- pre-menopausal females

. body mass index ≥ 40 kg/m2

- with stable weight in the three month before the study

- scheduled for bariatric surgery and in whom weight loss was clinically advisable before surgery

- non-smokers or smoking less than 5 cigarettes per day

Exclusion Criteria:

- pregnancy

- ischaemic heart disease

- cardiac failure

- high blood pressure requiring drug treatment

- tachyarrhythmia

- sick sinus syndrome

- atrioventricular block

- two-bundle ventricular block

- cerebrovascular diseases

- occlusive peripheral artery disease

- renal failure

- current treatment with drugs that might affect metabolic rate (e.g. β-adrenergic blockers, thyroid hormones).

This is a double-bling, parallel group design study. Patients will be randomised to 30-day treatment with either ephedrine + caffeine (EC) at the dosage of 200/20mg t.i.d. or placebo. The EC administration starts with an initial dose of 100/10 mg t.i.d. for the first week and then proceeds with the full dose of 200/20 mg t.i.d. The study pills (active drug and placebo) will be prepared by the Hospital's Pharmacy and coded with number corresponding to randomisation list.

Randomisation lists will be prepared by the Statistic consultant of the Istituto Auxologico Italiano who will have no direct contact with patients. Every four patients two will be allocated to EC and two to placebo. Since diabetes might in theory affect the extent of adrenergic response, randomisation list will be separate for diabetic and non-diabetic patients (diabetes diagnosed by oral glucose tolerance test at baseline using American Diabetes Association criteria). We aim at recruiting between 12 and 20 patients over a 12-month period. A dedicated clinical research fellow will be appointed for the study.

There are currently no restrictions to prescription of caffeine and ephedrine as galenic formulations by the Italian Ministry of Health, and the same dosage of EC used as active drug in the present study is currently registered and used for obesity treatment in Denmark (tradename Letigen).

Resting energy expenditure will be measured by indirect calorimetry at baseline and at the end of the study. This is a non-invasive determination requiring the patient to rest awake under a canopy for about 20 minutes while oxigen consumption and carboxy dioxide production is measured.

During the treatment period all patients are fed a hypocaloric diet (total energy content of ~70% of energy expenditure, as measured by indirect calorimetry), and containing 20% proteins, 55% carbohydrates, 25% fat half of which was monounsaturated, and 35 g/day fibres.

Due to the adrenergic properties of EC, in order to evaluate drug safety and early diagnose possible detrimental effect on cardiac function, the blood pressure will be measured three times a day; both electrocardiography and echocardiography will be recorded at baseline and every week.

Antropometric measurements (weight, height, waist circumference, hip circumference) and the following blood tests (in addition to routine blood panel for obese patients at our Institution, which includes oral glucose tolerance test) will be carried out at baseline and following the 30-days study period:

- fasting glucose
- fasting insulin
- fasting free fatty acids
- fasting glycerol
- plasma noradrenalin
- thyroid function (TSH, T3, T4)

After diet and drug period, the patients will be transferred from Piancavallo to the Department of Surgery (Molinette Hospital, Turin) for the bariatric surgery. The drug treatment (EC and placebo) will stopped the day before surgical intervention. Small biopsies of rectus abdominis and of subcutaneous and omental adipose tissues will be taken during surgery, immediately frozen in liquid nitrogen, and stored at -80°C for subsequent analysis. This procedure is judged to minimally affect operating times and does not carry additional risk to the patients.

Blood samples will be analysed at the Clinical Chemistry and Pathology Lab at Piancavallo. Data analysis will be carried out at the Clinical Nutrition Laboratory of the Istituto Auxologico Italiano. UCP3S and UCP3L mRNA levels will measured by quantitative polymerase chain reaction at the Department of Pharmacology, University of Milan. Presence of beta-3 adrenoceptor in adipose tissue will be evaluated by at the Department of Human Anatomy, University of Ancona using state-of-the art techniques.

**References**

1. Astrup A, Toubro S, Cannon S, Hein P, Madsen J. Thermogenic, metabolic, and cardiovascular effects of a sympathomimetic agent, ephedrine. Curr Ther Res 1990;48:1087–100.

2. Dulloo AG, Seydoux J, Girardier L. Peripheral mechanisms of thermogenesis induced by ephedrine and caffeine in brown adipose tissue. Int J Obes 1991;15:317–26.

3. Bukowiecki L, Jahjah L, Follea N. Ephedrine, a potential slimming drug, directly stimulates thermogenesis in brown adipocytes via beta-adrenoceptors. Int J Obes 1982;6:343–50.

4. Astrup A, Toubro S. Thermogenic, metabolic, and cardiovascular responses to ephedrine and caffeine in man. Int J Obes 993;17(suppl):41S–3S.

5. Dulloo AG, Seydoux J, Girardier L. Potentiation of the thermo- genic antiobesity effects of ephedrine by dietary methylxanthines: adenosine antagonism or phosphodiesterase inhibition? Metabolism 1992;41:1233–41.

6. Dulloo AG, Miller DS. The thermogenic properties of ephedrine/methylxanthine mixtures: human studies. Int J Obes 1986;10:467–81.

7. Toubro S, Astrup A, Breum L, Quaade F. The acute and chronic effects of ephedrine/caffeine mixtures on energy expenditure and glucose metabolism in humans. Int J Obes 1993;17(suppl):73S–7S.

8. Daly PA, Krieger DR, Dulloo AG, Young JB, Landsberg L. Ephedrine, caffeine and aspirin: safety and efficacy for treatment of human obe- sity. Int J Obes 1993;17(suppl):73S–8S.

9. Breum L, Pedersen JK, Ahlstrøm F, Frimodt-Møller J. Comparison of an ephedrine/caffeine combination and dexfenfluramine in the treatment of obesity. A double-blind multi-centre trial in general practice. Int J Obes 1994;18:99–103.

10. Klaus S, Casteilla L, Bouillaud F, Ricquier D. The uncoupling protein UCP: a membraneous mitochondrial ion carrier exclusively expressed in brown adipose tissue. Int J Biochem. 1991;23:791-801.

11. Grujic D, Susulic VS, Harper ME, Himms-Hagen J, Cunningham BA, Corkey BE, Lowell BB. Beta3-adrenergic receptors on white and brown adipocytes mediate beta3-selective agonist-induced effects on energy expenditure, insulin secretion, and food intake. A study using transgenic and gene knockout mice. J Biol Chem. 1997;272:17686-93.

12. Vidal-Puig A, Solanes G, Grujic D, Flier JS, Lowell BB. UCP3: an uncoupling protein homologue expressed preferentially and abundantly in skeletal muscle and brown adipose tissue. Biochem Biophys Res Commun. 1997 Jun 9;235(1):79-82.

13. Millet L, Vidal H, Andreelli F, Larrouy D, Riou JP, Ricquier D, Laville M, Langin D. Increased uncoupling protein-2 and -3 mRNA expression during fasting in obese and lean humans. J Clin Invest. 1997 Dec 1;100(11):2665-70.
